# Reduced Expression of Genes Regulating Cohesion Induces Chromosome Instability that May Promote Cancer and Impact Patient Outcomes

**DOI:** 10.1038/s41598-020-57530-9

**Published:** 2020-01-17

**Authors:** Tarik R. Leylek, Lucile M. Jeusset, Zelda Lichtensztejn, Kirk J. McManus

**Affiliations:** 1 Department of Biochemistry & Medical Genetics, University of Manitoba, Winnipeg, Manitoba R3E 0J9 Canada; 2 Research Institute in Oncology & Hematology, CancerCare Manitoba, Winnipeg, Manitoba R3E 0V9 Canada

**Keywords:** Cancer genetics, Cancer imaging

## Abstract

Chromosome instability (CIN), or continual changes in chromosome complements, is an enabling feature of cancer; however, the molecular determinants of CIN remain largely unknown. Emerging data now suggest that aberrant sister chromatid cohesion may induce CIN and contribute to cancer. To explore this possibility, we employed clinical and fundamental approaches to systematically assess the impact reduced cohesion gene expression has on CIN and cancer. Ten genes encoding critical functions in cohesion were evaluated and remarkably, each exhibits copy number losses in 12 common cancer types, and reduced expression is associated with worse patient survival. To gain mechanistic insight, we combined siRNA-based silencing with single cell quantitative imaging microscopy to comprehensively assess the impact reduced expression has on CIN in two karyotypically stable cell lines. We show that reduced expression induces CIN phenotypes, namely increases in micronucleus formation and nuclear areas. Subsequent direct tests involving a subset of prioritized genes also revealed significant changes in chromosome numbers with corresponding increases in moderate and severe cohesion defects within mitotic chromosome spreads. Collectively, our clinical and fundamental findings implicate reduced sister chromatid cohesion, resulting from gene copy number losses, as a key pathogenic event in the development and progression of many cancer types.

## Introduction

The International Agency for Research on Cancer estimated that in 2018, ~18.1 million individuals were newly diagnosed with cancer throughout the world, while an additional ~9.6 million succumbed to the disease^1^. Despite improved screening efforts, many cancers are still diagnosed at late stages (III and IV) when chemotherapy may be the only therapeutic option. Unfortunately, most late stage cancers are fatal and thus there is a critical need for a greater understanding of the pathogenic origins of cancer, so that novel precision medicine strategies can be devised to combat those origins.

Genome instability is defined by a high mutation frequency that includes nucleic acid sequence changes, chromosomal rearrangements and aneuploidy. Genome instability is a key driver of cancer pathogenesis and is estimated to occur in ~95% of all cancers^2–4^. Although there are many aberrant pathways giving rise to genome instability (reviewed in^5^), chromosome instability (CIN) is emerging as a central tenet in the development and progression of many cancer types^6^. CIN is defined as an increase in the rate at which whole chromosomes, or large parts thereof are gained or lost^7^, and is synonymous with cell-to-cell heterogeneity. Thus, to accurately assess CIN mandates the use of quantitative approaches capable of assessing either the rate of chromosomal changes over time, or cell-to-cell heterogeneity in CIN phenotypes at a single time point^8–10^. In general, CIN can be categorized into two subtypes: 1) numerical CIN, associated with changes in chromosome numbers; and 2) structural CIN, associated with gross chromosomal rearrangements, gene amplifications and deletions. As CIN promotes continual changes in chromosome and genetic complements, it is a key driver of genetic, cellular and intratumoural heterogeneity, and thus, cell-to-cell heterogeneity in chromosome complements is synonymous with CIN^6,11^. Although CIN is associated with cellular transformation, enhanced metastatic potential, multi-drug resistance and poor patient prognosis^12–16^, the mechanisms causing CIN remain poorly understood. In fact, cross-species approaches predict up to 2,300 CIN genes (*i.e*. those whose diminished expression induces CIN) exist in humans^17^; however, fewer than 150 have been identified and validated to date^17,18^. Of those identified, many encode functions within DNA replication, DNA repair, centrosome dynamics and chromosome segregation.

Recently, a body of evidence has begun to emerge implicating aberrant mitotic sister chromatid cohesion as a pathogenic event underlying CIN and tumour development^8^ (reviewed in^19^). Sister chromatid cohesion is regulated by a quaternary ring-like structure termed cohesin that is comprised of SMC1A (Structural Maintenance of Chromosomes 1 A), SMC3, RAD21 and one of STAG1, STAG2 or STAG3 (Stromal Antigen 1, -2 and -3). Conceptually, cohesin functions by tethering nascently synthesized sister chromatids together to prevent premature chromosome segregation from occurring (reviewed in^20,21^). Cohesin is first loaded onto chromosomes in G1 by the cohesion loaders, MAU2 and NIPBL, while the establishment of cohesion occurs in S-phase through the activities of ESCO1 and ESCO2. In humans and as cells enter into mitosis, cohesion is lost along the length of the chromosome arms, but is retained at the primary constriction (centromere) until RAD21 is cleaved and cells enter anaphase to initiate chromosome segregation. Previously, we determined that four cohesion genes, *SMC1A*, *SMC3*, *NIPBL* and *STAG3* are somatically mutated in colorectal cancer^8^. We further showed that reduced expression of *SMC1A* and *STAG2* induced cohesion defects leading to CIN^8^. Collectively, these data suggest reduced cohesion gene expression and defective sister chromatid cohesion as pathogenic events in cancer; however, a comprehensive assessment of 10 genes encoding key functions related to cohesion genes (*i.e*. cohesin, cohesion loaders and establishers) has never been performed. Moreover, recent evidence suggests many genes have divergent roles within the cell^22–25^, and thus their individual impacts on CIN and cancer remain largely unknown.

In this study, we sought to gain insight into the potential pathogenic relationship between reduced cohesion gene expression, CIN and cancer. Using cancer patient datasets, we determined that all 10 cohesion genes exhibit frequent deletions in many cancer types and that reduced expression corresponds with worse patient survival. Next, we employed single cell quantitative imaging microscopy (scQuantIM) in two karyotypically stable cell lines to show that reduced expression of each gene induced cell-to-cell heterogeneity and increases in two CIN-associated phenotypes. Subsequent direct tests involving a subset of genes also revealed significant changes in chromosome numbers that corresponded with increases in cohesion defects, as measured by primary constriction gaps (PCGs) within mitotic chromosome spreads. Thus, our fundamental and clinical findings show that reduced cohesion gene expression induces CIN, and is associated with worse patient outcomes in cancer. Collectively, these findings implicate reduced cohesion, stemming from gene copy number losses, as a key etiological events in the development and progression of many cancer types.

## Results

### Cohesion genes exhibit copy number losses and poor patient survival in cancer

**Figure 1:**
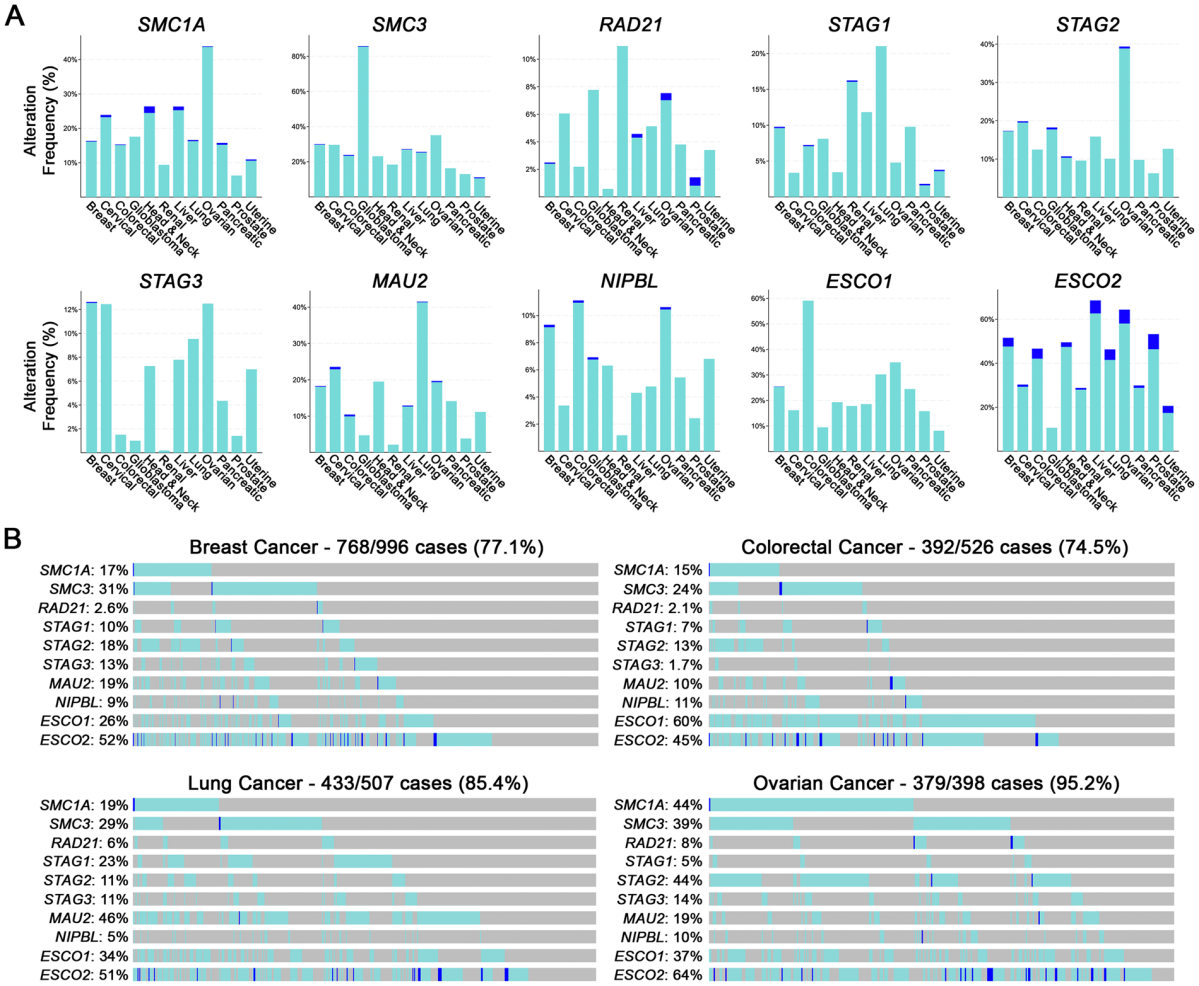
Cohesion gene copy number losses occur frequently in multiple cancer types. (**A**) Frequency of cohesion gene copy number losses in 12 common cancer types^27^. Shallow and deep deletions are shown in aqua and blue, respectively. (**B**) Cumulative frequency of copy number losses (shallow and deep deletions) for all cohesion genes in breast (77%), colorectal (75%), lung (85%) and ovarian (95%) cancers^26,27^.

To determine the potential impact that reduced cohesion gene expression may have in cancer, we focused our attention on 10 genes with key roles in sister chromatid cohesion, including those that encode; 1) the cohesin complex (*SMC1A*, *SMC3*, *RAD21*, *STAG1*, *STAG2* and *STAG3*); 2) cohesin loaders (*MAU2* and *NIPBL*); and 3) proteins required for the establishment of cohesion (*ESCO1* and *ESCO2*). Gene copy number alterations were assessed for all 10 genes in patient datasets derived from The Cancer Genome Atlas (TCGA) network (see Table S1 for genomic features)^26,27^. As shown in Fig. 1A, TCGA data reveal that each gene exhibits copy number losses (shallow and deep deletions) across 12 common cancer types. Furthermore, Fig. 1B shows that the collective frequency of shallow and deep deletions ranges from 75% in colorectal cancer to 95% in ovarian cancer, suggesting reduced expression may be a pathogenic event in cancer. In agreement with this possibility, reduced mRNA expression of each gene is associated with significant decreases in overall patient survival in various cancer types, including colorectal, cervical, glioma and ovarian cancers (Fig. S1). Collectively, these data are consistent with gene copy number deletions and reduced expression being pathogenic events in many cancer types; however, the mechanism linking copy number losses with cancer remains to be determined.

### Reduced cohesion gene expression induces increases in micronucleus formation and nuclear areas in HCT116 and hTERT cells

**Figure 2:**
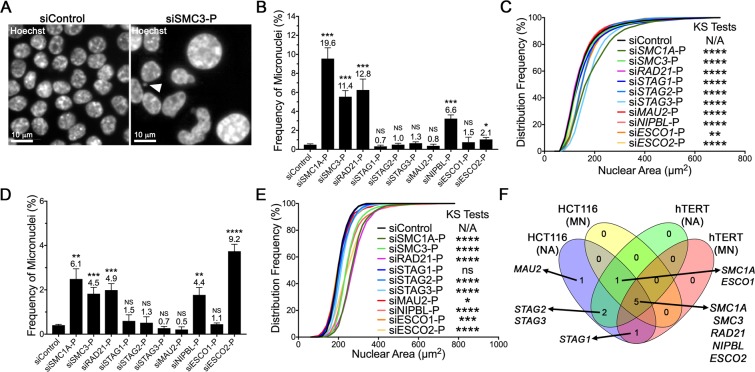
Cohesion gene silencing corresponds with increases in micronucleus formation and nuclear areas. (**A**) Representative high-resolution images highlighting a micronucleus (arrowhead) and the type of nuclear area heterogeneity induced following cohesion gene silencing (siSMC3-P; right) relative to siControl (left) in HCT116 cells. Note the scale bars are identical. (**B**) Bar graph presenting the mean frequency of micronuclei ± standard deviation (SD) following gene silencing relative to siControl in HCT116 cells. The mean fold increase in micronucleus formation relative to siControl is presented above each bar. Student’s *t*-tests comparing the mean frequency of micronuclei relative to siControl is as indicated (not significant [NS], p-value > 0.05; *p-value <0.05; ***p-value <0.001). (**C**) Cumulative nuclear area distribution frequencies following silencing of all 10 cohesion genes in HCT116 cells relative to siControl. Two-sample KS tests reveal statistically significant increases in nuclear area distributions relative to siControl (N/A, not applicable; **p-value <0.01; ****p-value <0.0001). (**D**) Bar graph showing changes in micronucleus formation following cohesion gene silencing in hTERT cells, with the mean fold increase and statistical significance indicated (Student’s *t*-tests; NS, p-value > 0.05; **p-value <0.01; ***p-value <0.001; ****p-value <0.0001). (**E**) Cohesion gene silencing induces significant increases in nuclear area distribution frequencies in hTERT cells (N/A, not applicable; ns, not significant [p-value > 0.05]; *p-value <0.05; ***p-value <0.001; ****p-value <0.0001). (**F**) Venn diagram presenting the results of the micronucleus formation and nuclear area assays conducted in HCT116 and hTERT cells. Five genes, *SMC1A, SMC3, RAD21, NIPBL* and *ESCO2* screened positive in both assays in both cell lines.

To determine the impact that reduced expression of all 10 cohesion genes has on CIN, a scQuantIM screen was performed, in which each gene was independently silenced and assessed for changes in two CIN-associated phenotypes (micronucleus formation and changes in nuclear area; Fig. 2A) in a karyotypically stable cell line, HCT116, which has been used extensively in CIN studies^8,18,28–31^. Conceptually, micronuclei are extranuclear bodies found outside of the primary nucleus and are hallmarks of CIN^32–34^, while changes in nuclear areas are associated with changes in DNA/chromosome complements^18,31^. Figure 2B shows the most profound increases in micronucleus formation occurred following silencing of *SMC1A*, *SMC3* and *RAD21*, (11.4- to 19.6-fold relative to siControl), with intermediate increases occurring following *ESCO2* or *NIPBL* silencing (2.1- to 6.6-fold) and no significant changes observed following *STAG1*, *STAG2*, *STAG3, MAU2* or *ESCO1* silencing (0.7- to 1.5-fold) (Table S2). The stronger phenotypes associated with *SMC1A*, *SMC3* and *RAD21* silencing likely reflect their central roles in cohesin, while the lack of phenotypes observed following *STAG1*, *STAG2* or *STAG3* silencing likely reflects their abilities to functionally compensate for one another^24,35,36^. Further, the observation that *ESCO2* silencing induced stronger phenotypes than *ESCO1* agrees with recent work uncovering distinct functions for ESCO1 and ESCO2 during cell cycle progression^22^. Next, scQuantIM was performed to determine the impact reduced expression has on nuclear areas. In general, gene silencing induced increases in cell-to-cell heterogeneity that two-sample Kolmogorov-Smirnov (KS) tests revealed corresponded with significant increases in nuclear areas distribution frequencies for all 10 genes (Fig. 2C; Table S3). Collectively, these findings show that reduced expression of cohesion genes is associated with increases in micronucleus formation and/or increases in nuclear areas in HCT116 cells.

To assess the conserved nature of these above findings and determine whether they are independent of cell type, similar experiments were performed in hTERT cells, a karyotypically stable, diploid fibroblast cell line that has been used in similar CIN-based studies^18,29,30^. In agreement with the above findings, gene silencing induced increases in micronucleus formation (Table S2), albeit to a lesser extent than in HCT116. As shown in Fig. 2D, large (4.4- to 9.2-fold) and statistically significant increases in micronucleus formation accompanied *SMC1A*, *SMC3*, *RAD21*, *NIPBL* and *ESCO2* silencing, whereas silencing the remaining genes had little to no impact (0.5- to 1.5-fold) on micronucleus formation. In agreement with the HCT116 screen, reduced cohesion gene expression generally corresponded with visual increases in nuclear areas (and changes in nuclear shapes) that coincided with statistically significant increases in cumulative distribution frequencies (Fig. 2E), with the exception of *STAG1* (Table S3). The combined results from HCT116 and hTERT (Fig. 2F) identify the three non-redundant central cohesin complex members (*SMC1A*, *SMC3* and *RAD21*), a cohesion loader (*NIPBL*) and an establisher of cohesion (*ESCO2*) as significant and strong candidates to pursue in subsequent validation work.

### SMC3 silencing induces significant changes in micronucleus formation and nuclear areas in HCT116 and hTERT cells

**Figure 3:**
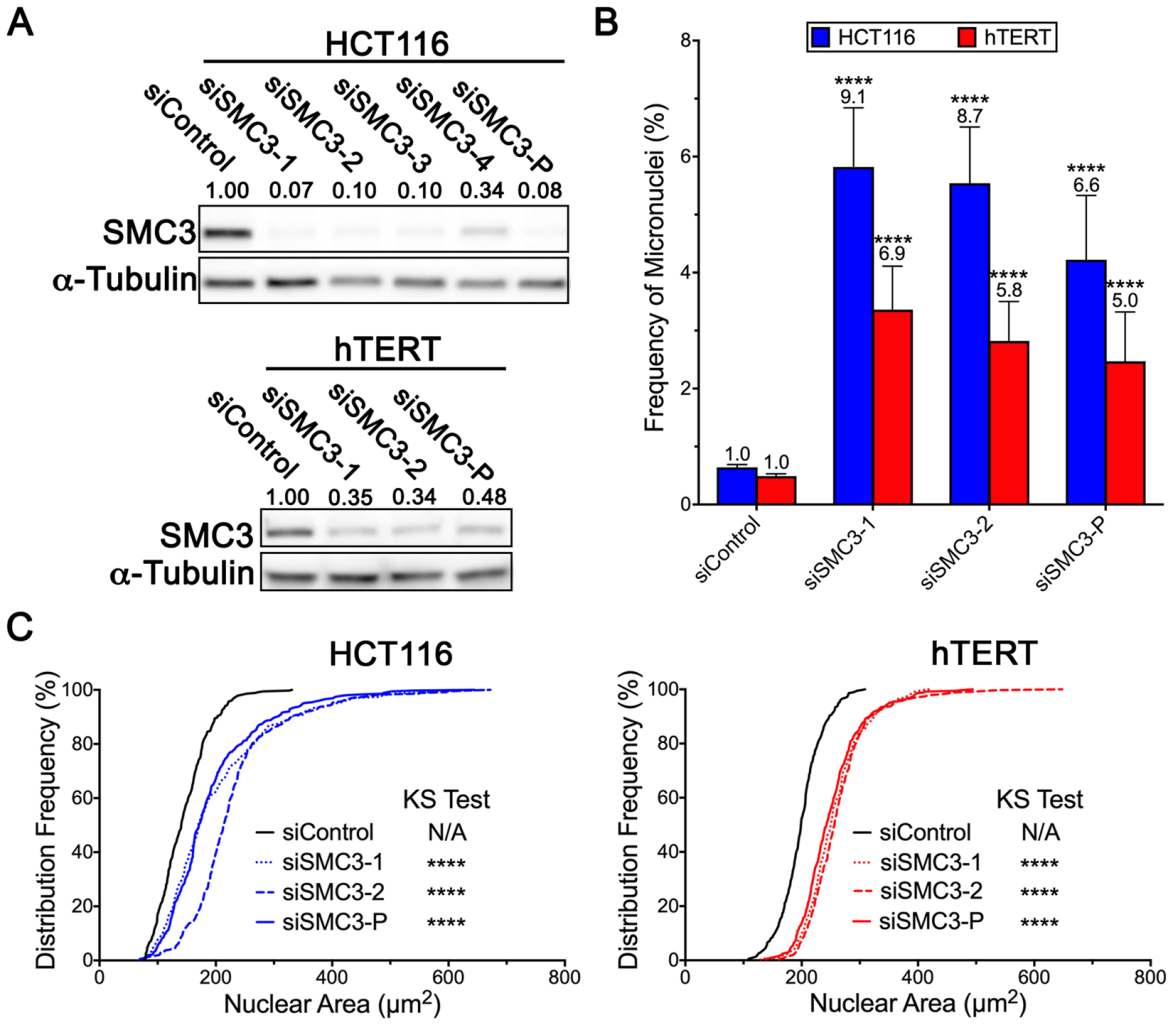
*SMC3* silencing induces CIN phenotypes in HCT116 and hTERT cells. (**A**) Representative western blots depicting the level of *SMC3* silencing induced in HCT116 and hTERT cells 96 h post-silencing with individual (siSMC3-1, -2, -3 and -4) and pooled (siSMC3-P) siRNA duplexes; α-Tubulin is the loading control. Semi-quantitative analyses were performed where SMC3 levels were normalized to the corresponding loading control and are presented relative to siControl (1.00). (**B**) Individual and pooled *SMC3* silencing induces significant increases in micronucleus formation relative to siControl in both HCT116 and hTERT cells (Student’s *t*-tests; ****p-value <0.0001). (**C**) Reduced *SMC3* expression corresponds with significant increases in nuclear area distribution frequencies in both HCT116 (left) and hTERT (right) cells (KS test; N/A, not applicable; ****p-value <0.0001).

*SMC3* was purposefully selected for subsequent validation as gene silencing induced reproducible and strong aberrant phenotypes in both HCT116 and hTERT cells, and we have previously established *SMC1A* as a CIN gene^8,31^. To confirm the results above are due to reduced *SMC3* expression, both individual and pooled siRNA duplexes were employed in a similar series of experiments; however, it was first necessary to establish the silencing efficiencies of the siRNAs employed. As shown in Fig. 3A, SMC3 levels were reduced to ~7–34% of endogenous levels in HCT116 using the individual duplexes (siSMC3-1, -2, -3 or -4), while the pooled approach (siSMC3-P) reduced expression to ~8%. Overall, the two most efficient silencing duplexes (siSMC3-1 and -2) along with the pool were employed in all subsequent work and were confirmed to reduce SMC3 levels to ~34-48% within hTERT cells. In agreement with the initial screen, *SMC3* silencing corresponded with significant increases in micronucleus formation (Fig. 3B), with the greatest increases occurring within HCT116 cells (Table S4). *SMC3* silencing also induced significant increases in nuclear areas within HCT116 and hTERT cells (Fig. 3C; Table S5). Collectively, these data validate the results of the initial screen and show that *SMC3* silencing with either individual or pooled siRNA duplexes induces similar phenotypes.

### Reduced cohesion gene expression induces numerical CIN and increases in moderate and severe cohesion defects

**Figure 4:**
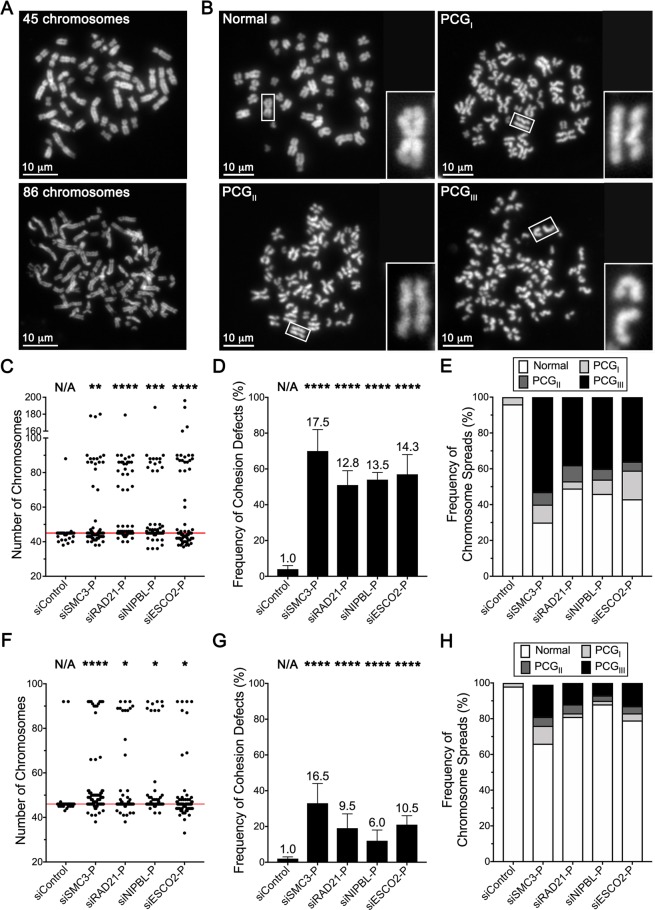
Reduced expression of *SMC3*, *RAD21*, *NIPBL* and *ESCO2* corresponds with increases in numerical CIN and cohesion defects. (**A**) Representative mitotic chromosome spreads exhibiting the modal number of 45 chromosomes (top; siControl) and increases in chromosome numbers (bottom; siSMC3-P) in HCT116 cells. (**B**) Representative micrographs of mitotic spreads from HCT116 showing normal primary constriction cohesion (top left) and the three categories of defective cohesion ranging from mild (PCG_I_; top right) to moderate (PCG_II_; bottom left) to severe (PCG_III_; bottom right). Each panel contains a bounding box for a region that is further magnified. (**C**) Dot plot showing significant increases in the distributions of chromosome numbers (numerical CIN) in HCT116 following gene silencing relative to siControl. Red line identifies modal number of 45 chromosomes. KS tests reveal significant changes in the chromosome number distributions relative to siControl (N/A, not applicable; **p-value <0.01; ***p-value <0.001; ****p-value <0.0001). (**D**) Bar graph showing significant increases in cohesion defects in HCT116 following gene silencing relative to siControl (Student’s *t*-tests; ****p-value <0.0001). (**E**) Bar graph showing the proportion of HCT116 cells with normal or defective (PCG_I_, PCG_II_ or PCG_III_) cohesion following gene silencing. (**F**) Dot plot showing significant increases in the distribution of chromosome numbers following gene silencing in hTERT cells. Red line identifies modal number of 46 chromosomes. (**G**) Gene silencing corresponds with significant increases in cohesion defects in hTERT cells. (**H**) Reduced gene expression corresponds with increases in PCG_I_, PCG_II_ and PCG_III_ categories in hTERT cells.

To identify the mechanism accounting for the CIN phenotypes induced above, four prioritized genes, *SMC3*, *RAD21*, *NIPBL* and *ESCO2* were silenced (Figs. S2 and S3) and mitotic chromosome spreads were generated and assessed for numerical CIN and sister chromatid cohesion defects (recall *SMC1A* is an established CIN gene^8,31^). Numerical CIN is assessed by enumerating chromosomes and statistically comparing differences in the overall distributions relative to siControl (Fig. 4A), while sister chromatid cohesion is visually assessed at the primary constriction as previously established (Fig. 4B)^8,37^. Briefly, as cells enter mitosis cohesion is normally lost along the length of the chromosome arms, but is retained at the primary constriction. Thus, cohesion defects are defined as a clear and distinct gap existing between sister chromatids at the primary constriction (*i.e*. PCGs). To further evaluate the types of cohesion defects occurring, chromosome spreads harboring PCGs were scrutinized and classified into one of three categories (Fig. 4B) based on the magnitude and prevalence of the phenotype; 1) PCG_I_, mild defect; 2) PCG_II_, moderate defect; and 3) PCG_III_, severe defect (see Methods for details)^8,37^.

*SMC3*, *RAD21*, *NIPBL* and *ESCO2* silencing in HCT116 induced large changes in chromosome numbers and distributions (Fig. 4C) that KS tests revealed are statistically distinct from siControl. Upon further inspection, the increases are most consistent with a tetraploidization event, and subsequent loss of a subset of chromosomes. With respect to cohesion, Fig. 4D shows that silencing corresponded with significant 12.8- to 17.5-fold increases in the frequency of cohesion defects, highlighting the pervasive nature of the cohesion defects that ranged from 51% (siRAD21-P) to 70% (siSMC3-P) of all spreads evaluated (Table S6). Remarkably, although gene silencing induced increases in all three PCG categories, it induced a high frequency of PCG_II_ and PCG_III_ events that were never observed within the siControl (Fig. 4E; Table S7). In summary, these data are consistent with reduced expression inducing numerical CIN arising from cohesion defects, particularly the PCG_II_ and PCG_III_ categories.

To confirm whether the above findings are independent of cell type, analogous experiments were performed in hTERT cells. In agreement with the HCT116 findings, *SMC3*, *RAD21*, *NIPBL* and *ESCO2* silencing induced significant, albeit less pronounced increases in chromosome number distributions relative to siControl (Fig. 4F). Similarly, silencing corresponded with 6.0- to 16.5-fold increases in overall cohesion defects (Fig. 4G; Table S6), the majority of which were PCG_II_ or PCG_III_ categories (Fig. 4H; Table S7). Collectively, these data confirm the results are celltype-independent and that reduced expression typically induces moderate and severe cohesion defects that drive numerical CIN.

## Discussion

In this study, we sought to explore the relationship between reduced cohesion expression and cancer by comprehensively assessing the impact reduced expression has on CIN, an enabling feature of cancer^4,38^. Using publicly available datasets, we first determined that cohesion genes exhibit frequent copy number losses in 12 cancer types, and that reduced expression correlated with worse patient survival. To explore the possibility that reduced expression may be a pathogenic event, we performed a scQuantIM screen in two karyotypically stable cell lines and showed that silencing promoted increases in micronucleus formation and/or nuclear areas. As reduced *SMC3*, *RAD21*, *NIPBL* and *ESCO2* expression induced strongest phenotypes in both cell lines, they were purposefully selected for in-depth analyses. As well as increases in micronucleus formation and nuclear areas, silencing also induced significant increases in chromosome numbers. To gain insight into the underlying mechanism, mitotic chromosome spreads were assessed for cohesion defects. Remarkably, reduced expression generated large increases in cohesion defects, most of which were moderate (PCG_II_) and severe (PCG_III_). Our current findings that gene silencing induces cohesion defects and numerical CIN agree with our previous work^8^, but extend those initial findings to include additional cohesin genes, cohesion loaders and genes required for the establishment of cohesion. This, coupled with our clinical assessments showing that copy number losses are associated with worse patient outcomes, firmly establish a link between aberrant cohesion and cancer and provides a mechanism to account for the observations of others, that reduced or mutant expression of specific genes correlates with worse patient outcomes^39–43^. Collectively, these findings demonstrate that reduced cohesion drives numerical CIN, which supports a causal link with cancer, and importantly, are consistent with reduced cohesion having pathogenic and clinical implications in many cancer types.

In general, gene silencing induced stronger and more prevalent phenotypes (*e.g*. increases in micronucleus formation and nuclear areas) in HCT116 relative to hTERT cells. Although the underlying mechanism(s) accounting for these differences remain unknown, there are several plausible explanations. First, the differences in the level of silencing achieved between the two lines may impact the magnitude of the aberrant phenotypes. For example, the greater silencing observed within the HCT116 cells is expected to result in stronger phenotypes (Figs. S2 and S3). Second, the distinct genetic contexts existing between the cell lines may be a contributing factor – HCT116 are a malignant, transformed colorectal cancer cell line with DNA mismatch repair defects (*MLH1* deficiency)^44^, while hTERT are a non-malignant, immortalized (human telomerase reverse transcriptase) cell line^45^. As HCT116 are mis-match repair deficient, they will accrue additional background mutations that may synergize with reduced cohesion gene expression to induce the more pronounced phenotypes observed. Finally, population doubling times may also have a role in the magnitude and/or prevalence of the aberrant phenotypes. HCT116 and hTERT have doubling times of ~22 h and 36 h, respectively, and thus over the 96 h time course of the experiments, HCT116 will undergo ~4.4 population doublings, whereas hTERT will undergo ~2.7. Population doublings are particularly critical as the cohesion defects are expected to induce CIN specifically during mitosis. Thus, HCT116 will have ~1.6-times more mitotic events, and thus more opportunities to induce CIN. Nevertheless, and irrespective of the underlying differences, both cell lines showed that reduced cohesion gene expression induced CIN and cohesion defects, confirming that the effects are cell type-independent.

Although cohesin functions to prevent premature sister chromosome segregation during mitosis, it also functions in organizing the 3D genome within interphase cells^46^, which has critical implications for DNA replication^47^, DNA repair^48^, gene transcription^49^ and telomere maintenance^50^. Thus, although our primary focus was on numerical CIN resulting from PGCs (*i.e*. cohesion defects) in mitotic cells, we cannot eliminate the possibility that cohesion defects may also adversely impact the 3D organization of the interphase nucleus and additional pathways that may induce numerical and/or structural CIN. For example, further scrutiny of the images revealed qualitative changes in nuclear shapes suggesting there may also be an impact on nuclear lamin expression (lamins A/C and/or B) that warrants further study. In addition, although we did observe increases in micronucleus formation that are suggestive of DNA double strand breaks for a subset of genes (*SMC1A*, *SMC3*, *RAD21*, *NIPBL* and *ESCO2*), we did not note a high frequency of DNA double strand breaks within the mitotic chromosome spreads, indicating the majority of these defects are numerical in nature.

Whilst the current study is focused on reduced expression of cohesion genes, it remains plausible that overexpression may also adversely impact cohesion and contribute to cancer development and progression. Interestingly, several genes, including *RAD21*, *STAG1*, *STAG3* and *NIPBL*, exhibit copy number gains across a spectrum of cancer types, and evidence from others show that increased expression is associated with worse outcome in certain cancer contexts and correlates with aneuploidy in Hodgkin lymphoma cell lines^37^. For example, Xu *et al*.^51^ showed that enhanced *RAD21* expression confers poor prognosis and drug resistance in high grade luminal, basal and HER2+ breast cancers, while Deb and colleagues^52^ showed that *RAD21* overexpression is predictive of poor prognosis in KRAS mutant colorectal cancers. In addition, enhanced *NIPBL* expression is associated with poor prognosis and chemotherapy resistance in non-small cell lung cancer^53^, while *SMC1A* overexpression contributes to colorectal cancer development in a mouse model^54^. While speculative, these findings, coupled with data from the current study strongly suggest that cohesion gene expression is normally tightly regulated, and that deviations, either through gene copy number losses or gains, may be etiological events contributing to the development and progression of cancer, although this remains to be empirically determined.

Overall, this study determined that the genes underlying sister chromatid cohesion are critical to preserve genome stability in humans and has identified a strong causal link between reduced expression and cancer. Indeed, the frequent gene copy number deletions and extensive array of mutations^55–57^ observed in many cancer types coupled with worse patient outcomes associated with low expression levels, strongly implicate reduced cohesion gene expression as key pathogenic events. In particular, reduced expression of *SMC3*, *RAD21*, *NIPBL* and *ESCO2* induced substantial cohesion defects and numerical CIN, and thus, may be significant, yet unappreciated drivers of cellular transformation and oncogenesis. As such, reduced expression of these genes and/or changes in nuclear areas (*i.e*. cell-to-cell heterogeneity) or micronucleus formation may hold diagnostic, prognostic or therapeutic value within the clinic; however, future studies are required to specifically investigate the correlation between changes in nuclear areas or micronucleus formation with treatment response and disease outcome for all relevant cancer types. In addition, the genomic alterations resulting in reduced expression may represent genetic susceptibilities that can be leveraged for highly specific and targeted killing of cancer cells (reviewed in^10,58–60^). Synthetic lethality is one such approach that is now showing potential within the clinic and may hold tremendous therapeutic potential is targeting the reduced expression of cohesion genes in many cancer types^61,62^.

## Methods

### Gene alterations and survival outcome analyses

Genomic and mRNA expression data generated by TCGA (https://portal.gdc.cancer.gov/)^27^ were used in all analyses. Publicly available data were extracted from 12 cancer types (breast, cervical, colorectal, glioblastoma, head & neck, renal, liver, lung, ovarian, pancreatic, prostate and uterine) using web-based analysis and visualization tools located at cBioPortal (www.cbioportal.org)^26^. User defined onco-query commands (HETLOSS and HOMDEL) were used to extract copy number variations for each query gene. Raw mRNA expression data and patient outcomes data were imported into Prism v7 (GraphPad). The thresholds used to distinguish between low and high mRNA expression was determined as described^63^, but in general, was selected as the mRNA expression level between the 20^th^ and 80^th^ percentiles that results in the lowest log-rank *p*-value in the survival analyses comparing patients with low or high mRNA expression levels. Kaplan-Meier curves were generated and log-rank tests comparing survival distributions were performed with *p*-values <0.05 deemed significant. All figures were assembled in Photoshop CS6 (Adobe).

### Cell culture

Two karyotypically stable human cell lines were employed, HCT116 (transformed epithelial colorectal cancer cell line with a modal number of 45 chromosomes) were purchased from the American Type Culture Collection (Rockville, MD), and hTERT (human telomerase immortalized fibroblast with a modal number of 46) were provided by Dr. C.P. Case (University of Bristol, Bristol, UK). Cells were grown in McCoy’s 5 A (HCT116) or Dulbecco’s modified Eagle’s medium (hTERT) containing 10% fetal bovine serum, and maintained at 37 °C in a humidified incubator (5% CO_2_). Cells were authenticated based on morphology, growth and spectral karyotyping as described^30^.

### Gene silencing and western blot analyses

Transient gene (*SMC1A*, *SMC3*, *RAD21*, *STAG1*, *STAG2*, *STAG3*, *MAU2*, *NIPBL*, *ESCO1* and *ESCO2*) silencing was performed by transfecting appropriate siRNA duplexes into cells using RNAiMax (Life Technologies; Burlington, Ontario) as detailed elsewhere^30^. Briefly, ON-TARGET*plus* (GE Dharmacon; Lafayette, Colorado) duplexes targeting distinct coding sequence regions within each gene were used either individually (siGENE-1, -2, -3 or -4) or as a Pool containing equal molar amounts of each individual siRNA (siGENE-P); *GAPDH* served as the negative control (siControl). In general, cells were seeded, permitted to attach and grow for 24 h prior to transfection with siRNAs. Gene silencing was assessed four days post-transfection by western blots, using the antibodies and dilutions indicated in Table 1. Briefly, proteins were electrophoresed, transferred to PVDF membranes that were subsequently cut horizontally to isolate the upper portion of the blot containing the protein of interest (*e.g.* SMC3), from the lower portion of the blot containing the loading control (α-tubulin). Semi-quantitative western blot analyses were performed to determine silencing efficiencies using ImageJ (densitometry), whereby the abundance of a particular protein (*e.g.* SMC3) was normalized to the corresponding loading control and is presented relative to the negative control (siControl), which is set to 1.00^30^.

**Table 1:** List of Antibodies and their Dilutions.

Target Protein	Species	Source	Catalog Number	Dilution
α-Tubulin	Mouse	Abcam	ab7291	1:20,000
Cyclophilin B	Rabbit	Abcam	ab16045	1:50,000
ESCO2	Rabbit	Abcam	ab220506	1:2,000
NIPBL	Rat	Abcam	ab106768	1:2,000
RAD21	Rabbit	Abcam	ab42478	1:5,000
SMC3	Rabbit	Abcam	ab9263	1:1,000
Anti-Mouse-HRP^a^	Goat	Jackson ImmunoResearch	115-035-146	1:10,000
Anti-Rabbit-HRP	Goat	Jackson ImmunoResearch	111-035-114	1:15,000
Anti-Rat-HRP	Goat	Abcam	ab9757	1:10,000

^a^HRP; horseradish peroxidase.

### Single cell quantitative imaging microscopy: nuclear areas and micronucleus formation

ScQuantIM was used to assess changes in nuclear areas and micronucleus formation following gene silencing as described previously relative to siControl, with *SMC1A* silencing serving as an established positive control that induces significant changes in both nuclear areas and micronucleus formation^8,31^. Briefly, cells were seeded into 96-well optically clear plates, permitted to attach (24 h) and transfected with siRNAs as detailed above. Four days post-transfection, cells were fixed (paraformaldehyde), counterstained (Hoechst 33342) and imaged using a Cytation 3 Cell Imaging Multi-Mode Reader (BioTek, Winooski, Vermont) equipped with a 16-bit charge-coupled device camera and a 20× Olympus LUCPLFLN lens (0.45 numerical aperture). A total of 9 non-overlapping (3 × 3 matrix) images per condition were acquired. Gen5 software (BioTek) was employed to extract nuclear areas from a minimum of 200 nuclei/condition using established size and fluorescence signal intensity exclusion filters to eliminate brightly stained bodies, including apoptotic and mitotic cells^28^. Nuclear area distributions were compared to siControl with two-sample KS tests and p-values <0.05 were considered significant. Next, an automated scQuantIM approach was used to determine the frequency of micronuclei within the same images assessed above. Briefly, images were segmented for two key image features, the primary (nuclear) mask and the secondary (cell body) mask, using Gen5 (BioTek). Spot detection was subsequently applied to identify micronuclei (small Hoechst-stained bodies) located outside the primary (nuclear) mask, but within the secondary (cell body) mask. To reduce type I and II errors and enhance image segmentation, several inclusion criteria/filters were employed: (1) a size filter was used to limit the analyses to micronuclei with a diameter ≤ 1/3 the of the primary nucleus (<5 μm); (2) a maximal mean fluorescence signal intensity (58,000 au) was used to exclude apoptotic and mitotic cells; and (3) and *x,y* image periphery exclusion filter (30 μm) to eliminate partial nuclei located along the image periphery. To address experimental reproducibility, all experiments were performed three times so that the mean frequency of micronuclei (%) was determined for each condition. Finally, Student’s *t*-tests were performed comparing the mean frequency of micronuclei for each gene relative to the siControl with a p-value <0.05 considered statistically significant. All descriptive statistics (number, mean, standard deviation), two-sample KS tests, Student’s *t*-tests and graphs were generated in Prism, while figures were assembled in Photoshop.

### Mitotic spreads and chromosome enumeration

Mitotic chromosome spreads were generated following gene silencing as described^30^. Chromosomes were manually enumerated from a minimum of 100 spreads/condition, and data were imported into Prism where descriptive statistics, graphs and KS tests were performed as described^29^.

### Cohesion assay

Cohesion assays were performed as described elsewhere^8,37^. Briefly, mitotic chromosome spreads were generated, counterstained with DAPI and chromosomes were visually inspected for the presence of cohesion defects at the primary constriction (*i.e*. centromere). Chromosome spreads displaying PCGs were classified into mild (PCG_I_), moderate (PCG_II_) or severe (PCG_III_) categories, based on the magnitude and prevalence of the defects. We define PCG_I_ as those spreads exhibiting mild cohesion defects in fewer than six chromosomes, PCG_II_ as those with moderate cohesion defects in six or more chromosomes, and PCG_III_ as spreads with severe cohesion defects in which any semblance of cohesion is lost and sister chromatids appear to be randomly distributed within the chromosome spread. All data were exported into Prism where descriptive statistics and graphs were generated.

## Supplementary information

Supplementary Information.

## Data Availability

Patient related data (Fig. 1) are based upon data generated by the TCGA Research Network and are available at https://www.cancer.gov/tcga. Data pertaining to the 10 cohesion related genes including specific genomic features are available in Supplementary Table S1. All descriptive statistics and statistical analyses presented in Figs. 2–4 are provided within Supplementary Tables S2–S7.
