# Supplementary material for: Reduced Expression of Genes Regulating Cohesion Induces Chromosome Instability that May Promote Cancer and Impact Patient Outcomes
Source: Sci Rep. 2020 Jan 17;10:592. doi: 10.1038/s41598-020-57530-9 (PMC6969069; doi:10.1038/s41598-020-57530-9)
Supplement: Supplementary file 1 — Supplementary Information. [file 41598_2020_57530_MOESM1_ESM.docx]

**Reduced Expression of Genes Regulating Cohesion Induces Chromosome Instability that may Promote Cancer and Impact Patient Outcomes**

Tarik R. Leylek^1^, Lucile M. Jeusset^1,2^, Zelda Lichtensztejn^2^ and Kirk J. McManus^1,2*^

^1^Department of Biochemistry & Medical Genetics, University of Manitoba,

Winnipeg, Manitoba, R3E 0J9, Canada

^2^Research Institute in Oncology & Hematology, CancerCare Manitoba,

Winnipeg, Manitoba, R3E 0V9, Canada

Running Title: Reduced cohesion expression induces CIN

***Address for Correspondence:**

Kirk J. McManus

Research Institute of Oncology and Hematology

ON6010 – 675 McDermot Avenue

Winnipeg, MB

R3E 0V9, CANADA

Phone: (204) 787-2833

Fax: (204) 787-2190

Email: Kirk.McManus@umanitoba.ca

Key Words: sister chromatid cohesion; cohesin; genome instability; chromosome instability; single cell quantitative imaging microscopy; cancer

**SUPPLEMENTARY MATERIALS**

**SUPPLEMENTARY FIGURES**


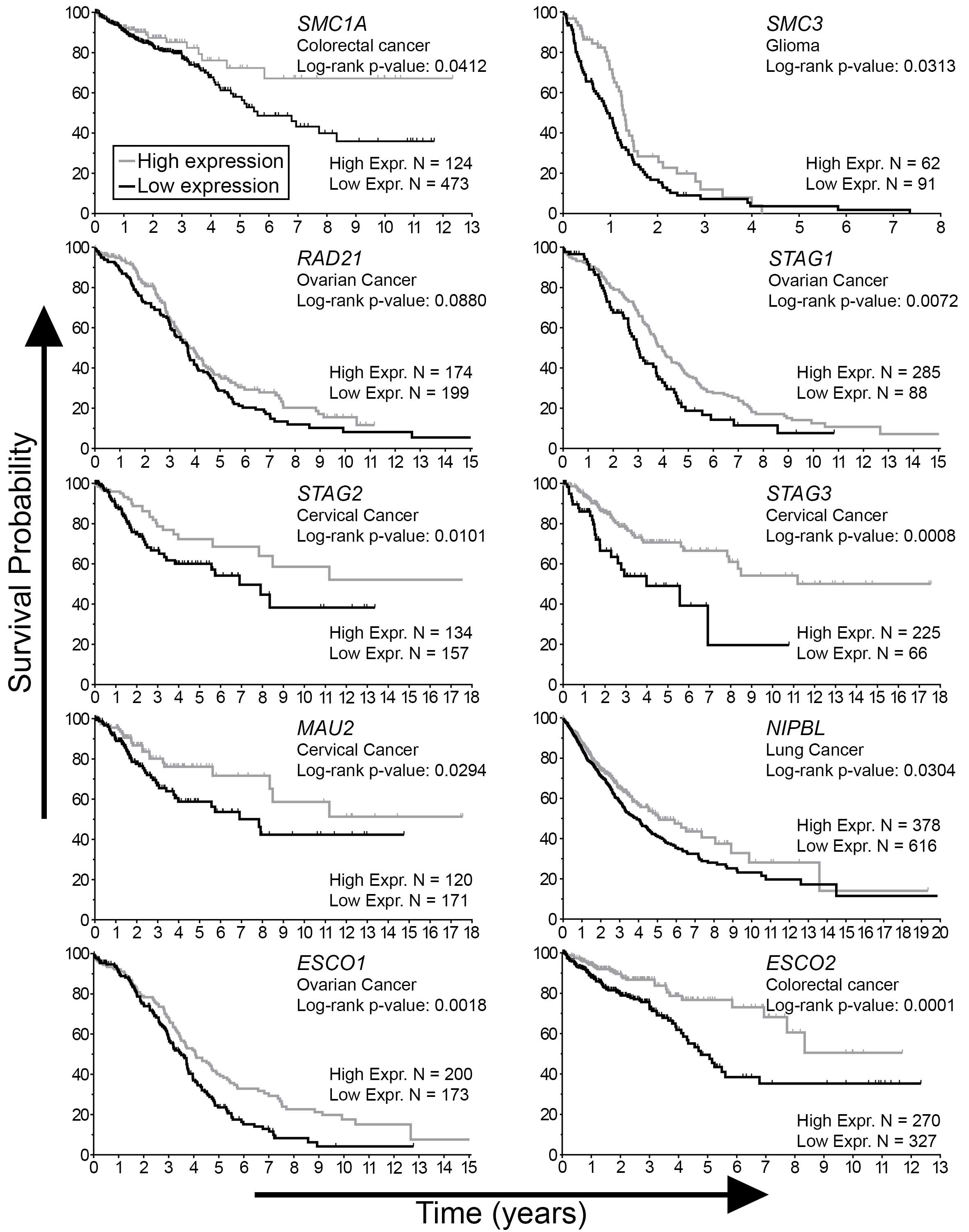


**Figure S1: Reduced cohesion expression is associated with worse patient survival.** Kaplan-Meier curves reveal low mRNA expression of cohesion genes is associated with worse overall patient survival in numerous cancer types [24,26].


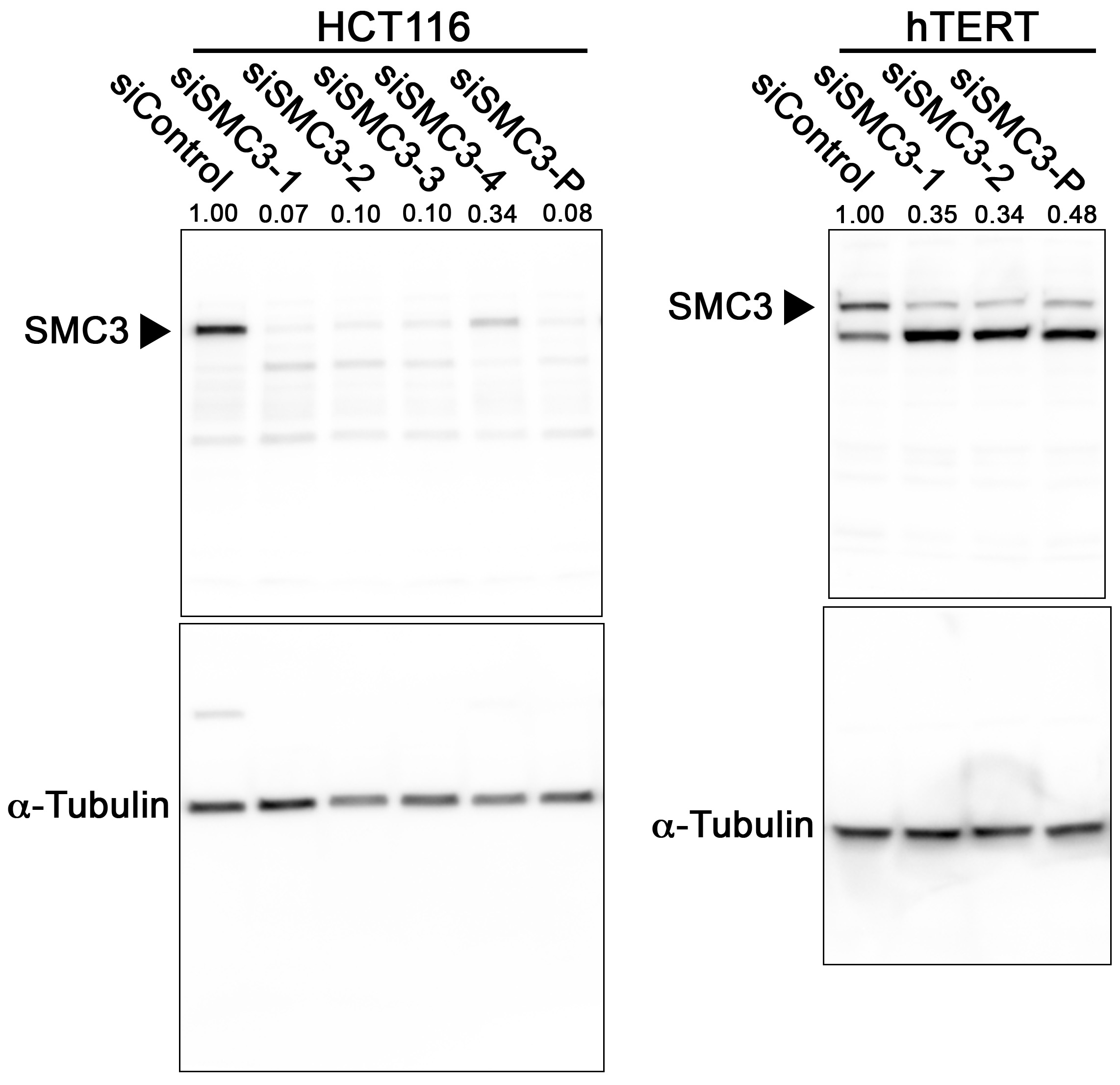


**Figure S2: Raw data for the western blots presented in Figure 3.**

Western blots of SMC3 (top) and α-tubulin (bottom) from HCT116 (left) and hTERT (right) cells. Densitometry analyses were performed using ImageJ. Each SMC3 value was first normalized to the corresponding loading control and is presented relative to the siControl, which is set to 1.00.


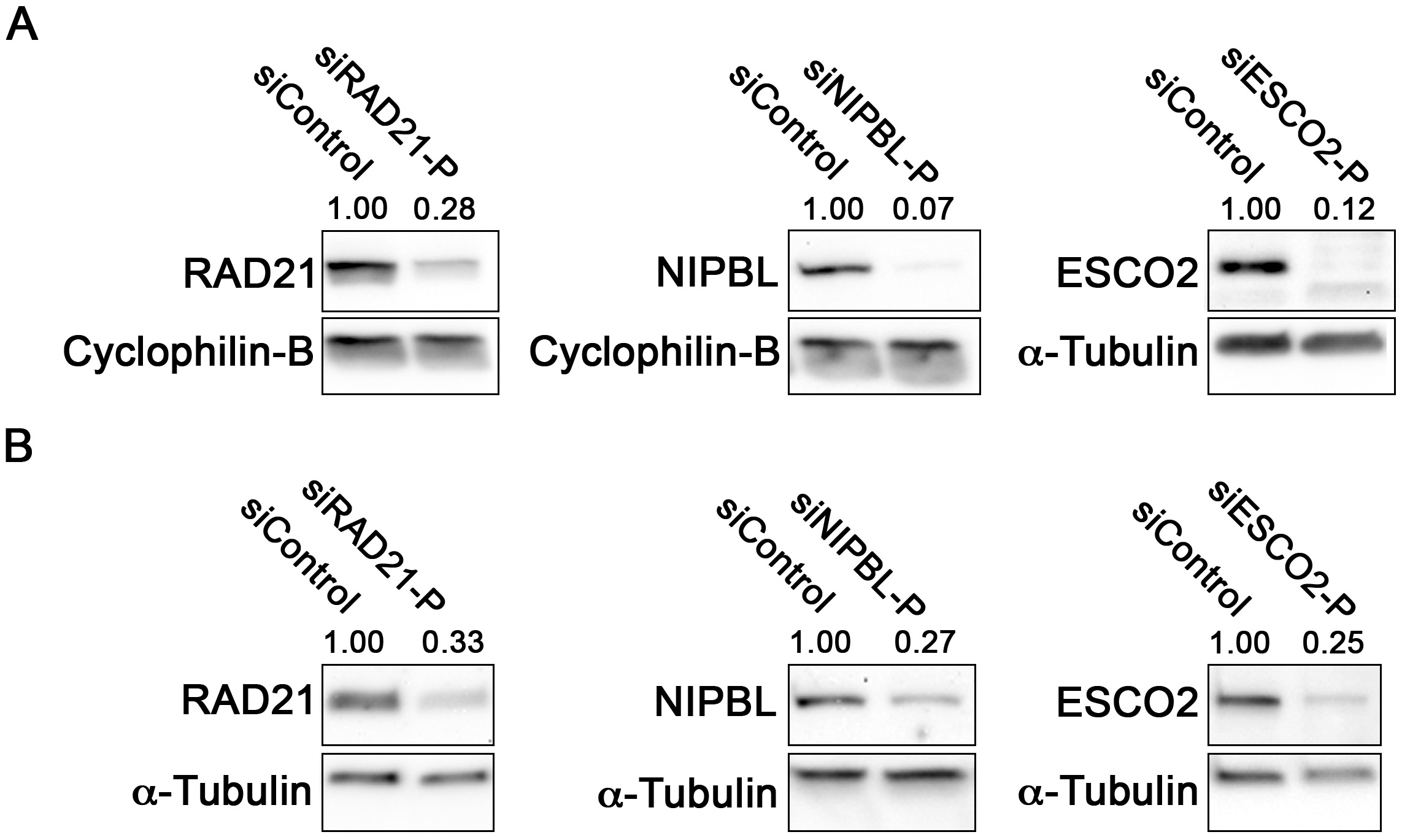


**Figure S3: Gene silencing efficiencies in HCT116 and hTERT cells.** (**A**) Semi-quantitative western blots presenting residual proteins levels following *RAD21* (left), *NIPBL* (middle) and *ESCO2* (right) silencing with pooled siRNA duplexes in HCT116 cells. Cyclophilin-B and α-Tubulin are loading controls. Residual protein levels are presented normalized to the respective loading control and are presented relative to siControl, which is set at 1.00. (**B**) Western blots showing residual RAD21, NIPBL and ESCO2 protein levels following silencing in hTERT cells.

**SUPPLEMENTARY TABLES**

**Table S1: Genomic features of cohesion-related genes**

**Accession Size^C^ Size**

**Gene Locus^A^ Number (mRNA)^B^ (Bases) (Amino Acids)**

*SMC1A* Xp11.22 NM_006306.3 48,580 1,233

*SMC3* 10q25.2 NM_005445.3 36,946 1,217

*RAD21* 8q24.11 NM_006265.2 28,933 631

*STAG1* 3q22.3 NM_005862.3 416,170 1,258

*STAG2* Xq25 NM_006603.4 462,453 1,231

*STAG2* 7q22.1 NM_012447.3 43,926 1,225

*MAU2* 19p13.11 NM_015329.4 37,938 613

*NIPBL* 5p13.2 NM_015384.4 189,655 2,804

*ESCO1* 18q11.2 NM_052911.3 71,604 840

*ESCO2* 8p21.1 NM_001017420.3 41,164 601

^A^Gene locus is based on Human Genome Organization (HUGO) Gene Nomenclature Committee (HGNC)

^B^GeneBank accession number (NCBI Reference Sequence)

^C^Gene size is based on human reference genome (GRch38/hg38)

**Table S2: Reduced cohesion gene expression induces increases in micronucleus formation in HCT116 and hTERT cells.**

**Cell Line Condition No. of N Mean ± SD^1^ Fold p-value^3^**

**Replicates Increase^2^**

HCT116 siControl 3 14241 0.488 ± 0.092 1.0 NA

siSMC1A-P 3 2648 9.554 ± 1.133 19.6 0.0002

siSMC3-P 3 2419 5.539 ± 0.647 11.4 0.0002

siRAD21-P 3 400 6.250 ± 1.148 12.8 0.0010

siSTAG1-P 3 4994 0.320 ± 0.119 0.7 0.1252

siSTAG2-P 3 6498 0.493 ± 0.145 1.0 0.9644

siSTAG3-P 3 3685 0.651 ± 0.142 1.3 0.1685

siMAU2-P 3 2955 0.372 ± 0.172 0.8 0.3619

siNIPBL-P 3 1916 3.236 ± 0.392 6.6 0.0003

siESCO1-P 3 2392 0.753 ± 0.529 1.5 0.4416

siESCO2-P 3 2767 1.048 ± 0.196 2.1 0.0109

hTERT siControl 3 5940 0.405 ± 0.037 1.0 NA

siSMC1A-P 3 1023 2.489 ± 0.464 6.2 0.0015

siSMC3-P 3 1576 1.825 ± 0.282 4.5 0.0010

siRAD21-P 3 553 1.989 ± 0.291 4.9 0.0007

siSTAG1-P 3 2345 0.597 ± 0.276 1.5 0.2975

siSTAG2-P 3 2347 0.511 ± 0.273 1.3 0.5390

siSTAG3-P 3 1859 0.269 ± 0.080 0.7 0.0554

siMAU2-P 3 2399 0.208 ± 0.123 0.5 0.0569

siNIPBL-P 3 1130 1.770 ± 0.335 4.4 0.0022

siESCO1-P 3 2452 0.449 ± 0.059 1.1 0.3340

siESCO2-P 3 1123 3.740 ± 0.317 9.2 <0.0001

^1^Mean micronucleus frequency ± standard deviation (SD)

^2^Fold increase in micronucleus formation relative to the corresponding siControl

^3^Student’s *t*-tests comparing mean micronucleus frequency to the corresponding siControl (N/A; not applicable). A p-value of <0.05 is considered statistically significant.

**Table S3: KS tests reveal significant differences in nuclear area distributions following silencing of cohesion genes in HCT116 and hTERT cells.**

**Percentile**

**Cell Line Condition N 25^th^ 50^th^ 75^th^ p-value^1^**

HCT116 siControl 5701 109 136 177.5 N/A

siSMC1A-P 2525 147 183 228 <0.0001

siSMC3-P 2429 141.5 174 222 <0.0001

siRAD21-P 1923 134 172 245 <0.0001

siSTAG1-P 5050 113 139 183 <0.0001

siSTAG2-P 6450 107 133 179 <0.0001

siSTAG3-P 3667 110 142 190 <0.0001

siMAU2-P 3392 129 157 195 <0.0001

siNIPBL-P 1895 145 181 223 <0.0001

siESCO1-P 2724 113 138 175 0.0039

siESCO2-P 2681 130 159 200 <0.0001

hTERT siControl 2094 183 206 234 N/A

siSMC1A-P 1014 240 273 309.3 <0.0001

siSMC3-P 1546 219 247 280 <0.0001

siRAD21-P 543 243 278 319 <0.0001

siSTAG1-P 2099 181 208 240 0.0914

siSTAG2-P 2190 189 215 250 <0.0001

siSTAG3-P 1786 197 223 254 <0.0001

siMAU2-P 1365 179 207 242 0.0249

siNIPBL-P 1071 218 249 287 <0.0001

siESCO1-P 2087 177 204 242 0.0004

siESCO2-P 858 207 253 308 <0.0001

^1^Two sample KS tests comparing cumulative nuclear area frequency distribution to the corresponding siControl (N/A; not applicable; ns, not significant [p-value >0.05]).

**Table S4: Reduced *SMC3* expression corresponds with significant increases in micronucleus formation in HCT116 and hTERT cells.**

**Cell Line Condition No. of N Mean ± SD^1^ Fold p-value^3^**

**Replicates Increase^2^**

HCT116 siControl 3 18273 0.64 ± 0.05 1.0 N/A

siSMC3-1 3 6647 5.82 ± 1.02 9.1 <0.0001

siSMC3-2 3 5823 5.54 ± 0.97 8.7 <0.0001

siSMC3-P 3 7012 4.22 ± 1.11 6.6 <0.0001

hTERT siControl 3 16253 0.49 ± 0.04 1.0 N/A

siSMC3-1 3 5548 3.36 ± 0.75 6.9 <0.0001

siSMC3-2 3 6132 2.82 ± 0.68 5.8 <0.0001

siSMC3-P 3 4809 2.47 ± 0.85 5.0 <0.0001

^1^Mean micronucleus frequency ± standard deviation (SD)

^2^Fold increase in micronucleus formation relative to the corresponding siControl

^3^Student’s *t*-tests comparing mean micronucleus frequency to the corresponding siControl (N/A; not applicable). A p-value of <0.05 is considered statistically significant.

**Table S5: *SMC3* silencing induces significant increases in nuclear area distributions in HCT116 and hTERT cells.**

**Percentile**

**Cell Line Condition N 25^th^ 50^th^ 75^th^ p-value^1^**

HCT116 siControl 350 112 141 174 N/A

siSMC3-1 350 132.8 170 242.5 <0.0001

siSMC3-2 350 181 213 252 <0.0001

siSMC3-P 350 138 171 219.3 <0.0001

hTERT siControl 350 177.8 199 220.3 N/A

siSMC3-1 350 223.8 250.5 282.3 <0.0001

siSMC3-2 350 228 257 285 <0.0001

siSMC3-P 350 216 243 277 <0.0001

^1^Two sample KS tests comparing cumulative nuclear area frequency distribution to the corresponding siControl (N/A; not applicable; ns, not significant [p-value >0.05]).

**Table S6: Reduced cohesion gene expression corresponds with significant increases in cohesion defects in HCT116 and hTERT cells.**

**Cell Line Condition N Mean ± SD^1^ Fold p-value^3^**

**Increase^2^**

HCT116 siControl 300 4 ± 2 1.0 N/A

siSMC3-P 300 70 ± 12 17.5 <0.0001

siRAD21-P 300 51 ± 8 12.8 <0.0001

siNIPBL-P 300 54 ± 4 13.5 <0.0001

siESCO2-P 300 57 ± 11 14.3 <0.0001

hTERT siControl 300 2 ± 1 1.0 N/A

siSMC3-1 300 33 ± 11 16.5 <0.0001

siSMC3-2 300 19 ± 8 9.5 <0.0001

siSMC3-P 300 12 ± 6 6.0 <0.0001

siESCO2-P 300 21 ± 5 10.5 <0.0001

^1^Mean frequency of cohesion defects ± SD

^2^Fold increase in cohesion defects relative to the corresponding siControl

^3^Student’s *t*-tests comparing mean cohesion defect frequency to the corresponding siControl (N/A; not applicable).

**Table S7: Reduced cohesion gene expression induces increases in primary constriction gaps in HCT116 and hTERT cells.**

**Frequency of PCG^1^ Defects (%) Fold**

**Cell Line Condition N PCG_I_ PCG_II_ PCG_III_ Total Increase^2^**

HCT116 siControl 300 4.0 0.0 0.0 4.0 1.0

siSMC3-P 300 10.0 7.0 53.0 70.0 17.5

siRAD21-P 300 4.0 9.0 38.0 51.0 12.8

siNIPBL-P 300 8.0 6.0 40.0 54.0 13.5

siESCO2-P 300 16.0 5.0 36.0 57.0 14.3

hTERT siControl 300 2.0 0.0 0.0 2.0 1.0

siSMC3-P 300 10.0 5.0 18.0 33.0 16.5

siRAD21-P 300 2.0 5.0 12.0 19.0 9.5

siNIPBL-P 300 2.0 3.0 7.0 12.0 6.0

siESCO2-P 300 4.0 4.0 13.0 21.0 10.5

^1^PCG; primary constriction gap. Mitotic chromosome spreads were visually assessed for the presence of mild (PCG_I_), moderate (PCG_II_) or severe (PCG_III_) cohesion defects.

^2^Fold increase in the frequency of PCG defects relative to siControl.
